# The molecular basis of targeting PFKFB3 as a therapeutic strategy against cancer

**DOI:** 10.18632/oncotarget.19513

**Published:** 2017-07-24

**Authors:** Luo Lu, Yaoyu Chen, Yu Zhu

**Affiliations:** ^1^ Department of Hematology, The First Affiliated Hospital of Nanjing Medical University, Jiangsu Province Hospital, Nanjing 210029, China

**Keywords:** PFKFB3, glycolysis, autophagy, solid tumors, hematologic malignancies

## Abstract

6-phosphofructo-2-kinase/fructose-2, 6-bisphosphatases (PFKFBs) are bifunctional enzymes which regulate the transformation between fructose-2, 6-bisphosphate (F2, 6BP) and fructose-6-phosphate (F6P) in the process of glucose metabolism. Among the four isozymes (PFKFB1-4), PFKFB3 has stronger kinase activity than phosphatase activity, resulting in the synthesis of F2, 6BP and the promotion of glycolysis. Additionally, PFKFB3 plays a key role in cell cycle regulation. It has been confirmed that PFKFB3 is upregulated in a variety of tumor cells, and inhibition of it results in suppression of the growth of tumor cells by downregulating the glycolytic flux. It is expected to release drug resistance and prevent disease progression by PFKFB3 inhibition. Recent studies have also shown that the efficacy of PFKFB3 inhibition in tumor cells is not only related to glycolysis, but also autophagy. Here, we have reviewed the biological characteristics of PFKFB3, the regulation pathway of glucose metabolism manipulated by PFKFB3, and other regulatory mechanisms in hematologic and non-hematologic malignant tumor cells.

## BACKGROUND

PFKFB is a member of the bifunctional enzyme family which are involved in both the synthesis and degradation of F2, 6BP. It is a critical regulatory molecule in glycolysis control [1]. The active center of its N-terminal region has a 6-phosphofructo-2-kinase activity that catalyzes the synthesis of F2, 6BP, and C-terminal domain of it has fructose-2, 6-biphosphatase activity that catalyzes the degradation of F2, 6BP. It acts as a regulator of cyclin-dependent kinase 1, linking glucose metabolism to cell proliferation and survival in tumor cells. Four isoforms, PFKFB1-4, was found to be encoded by PFKFB. Although 85% of core sequence of 2-Kase/2-Pase is coincident, different residues around the N-terminal, C-terminal variable region, and active site caused different functional characteristics. Owing to lacking a specific serine for phosphorylation, kinase activity of PFKFB3 was found to be 740-fold higher than phosphatase activity [2, 3]. PFKFB3 protein expression is found significantly higher in rapid proliferation cells [4], such as in solid tumors and hematologic malignancies [5, 6]. It can be regulated by numerous critical tumor-related genes and pathways. For instance, PFKFB3 gene promoter contains *HIF-1 (*hypoxia-inducible factor-1) binding sites and subsequently can be activated by *HIF-1* [7], MK2 (MAPK (mitogen-activated protein kinase)-activated protein kinase 2) activates PFKFB3 gene promoter region resulting in increased PFKFB3 transcription [8], and also loss of *PTEN* (phosphatase and tensin homolog) was found decreasing the degradation of PFKFB3 [9]. The strong kinase activity of PFKFB3 helps F2, 6BP synthesis, which is not only a glycolysis intermediate but also a key allosteric activator of phosphofructokinase-1 (PFK-1) [10], and subsequently increase the glycolysis flux. Cancer cells with high proliferation activity present an increased need for energy, but how the increased glycolysis are manipulated was not well understood and perhaps differ between types of tumors. As the regulation function of PFKFB3 in catalytic activity will be related to metabolic change in cells, PFKFB3 is supposed to be a critical factor in neoplastic transformation. Therefore, increasing numbers of researchers focused on the role of PFKFB3 in the regulation of tumor cell proliferation and metabolism.

### Molecular biological characteristics of PFKFB3

The *PFKFB3* gene is located in the chromosome 10p15.1 [11] and contains 19 regions that may encode exons, 15 of which are routinely expressed [12]. The 5′ promoter of this gene contains Sp (Specific protein) -1, AP (activator protein) -2 binding domains, HRE (hypoxia response element), and SRE (serum-response element). These specific binding regions play important roles in the regulation of glycolysis. For example, the *PFKFB3* gene can be activated by Sp-1 and AP-2 binding domains by phorbol esters and cAMP (cyclic-adenosine monophosphate)-dependent protein kinase signal activation. In hypoxic conditions, the expression of PFKFB3 can be induced by HIF-1 binding to HRE. Activated SRF (serum-response factor) can also bind to SRE and promote the expression of PFKFB3 [7, 13, 14].

### PFKFB3 and glucose metabolism in tumor cells

#### Glycolysis of tumor cells

Glycolysis is the first step of the glucose metabolism process in the cytoplasm of all biological cells regardless of aerobic or anaerobic environment. In this process, one glucose molecule transforms to two molecules of pyruvate after 10 steps of an enzymatic reaction. Under aerobic conditions, pyruvate is decomposed into acetyl-CoA and carbon dioxide (CO2). Then, acetyl-CoA enters the tricarboxylic acid cycle (TAC) and degrades into CO2 and hydrogen. Hydrogen binds to its carriers, nicotinamide adenine dinucleotide (NAD) and flavin adenine dinucleotide (FAD), forming NADH and FADH2, respectively. NADH and FADH oxidation lead to ATP production through the mitochondrial respiratory chain. In an anoxic environment, pyruvate is transformed into lactic acid, eventually generating ethanol and CO2 through anaerobic glycolysis. Anaerobic glycolysis is the original method of energy production. Although biological organisms retain this approach, the main pathway providing energy is the degradation of glucose by the TAC. Cells usually only initiate the anaerobic glycolysis pathway under hypoxic conditions. However, many tumor cells use this more primitive method as their main energy supply, which is called Warburg effect [15]. Even tumor cells with an abundant oxygen supply, such as lung carcinoma or leukemia cells, which are in direct contact with oxygen or oxygen-carrying blood fluid and have enough oxygen for oxidation, still use glycolysis as their main energy source. Some non-tumor cells also use this primitive method to burn glucose. It has recently been found that 85% of endothelial cells produce adenosine triphosphate (ATP) by anaerobic glycolysis, and blocking the anaerobic glycolysis pathway inhibits neovascularization [16], suggesting that the significance of anaerobic glycolysis for cells is not just for ATP production.

What is the significance of anaerobic glycolysis in cells? Is the Warburg effect only a concomitant phenomenon of rapid cell proliferation? Actually, anaerobic glycolysis has been proven to be critical for cell survival in several ways [17–21]: 1) ATP production occurs much more rapidly in anaerobic glycolysis than oxidation, which ensures high cellular proliferation flexibility. 2) Anaerobic glycolysis provides the raw materials for cell proliferation, which are intermediates for the synthesis of biological macromolecules, such as acetyl-CoA, NADPH (reduced form of Nicotinamide adenine dinucleotide phosphate), non-essential amino acids, and ribosomes. 3) By anaerobic glycolysis, cells generate energy more economically for environment adaption. In glucose sufficient circumstances, cells produce less ATP and simultaneously have lower enzyme consumption. 4) Cells avoid reactive oxygen species (ROS)-related damage via anaerobic glycolysis relative to oxidative phosphorylation. Meanwhile, tumor cells can synthesize NADPH with glucose, which plays an antioxidant role for further reducing intracellular ROS.

How is glycolytic flux regulated? One key step is the transformation of F6P to fructose-1, 6-bisphosphate (F1, 6BP) catalyzed by PFK-1, which is an irreversible biochemical reaction. The activity of PFK-1 is regulated by ATP, adenosine diphosphate (ADP), AMP, and F2, 6BP. PFK-1 activity is inhibited by increased intracellular ATP or citric acid, but this inhibition can be reversed by F2, 6BP through strongly reactivated PFK-1 [22, 23]. As mentioned before, PFKFB3 has strong kinase activity and promotes the synthesis of F2, 6BP, which subsequently activates PFK-1 and increases glycolysis.

**Figure 1:**
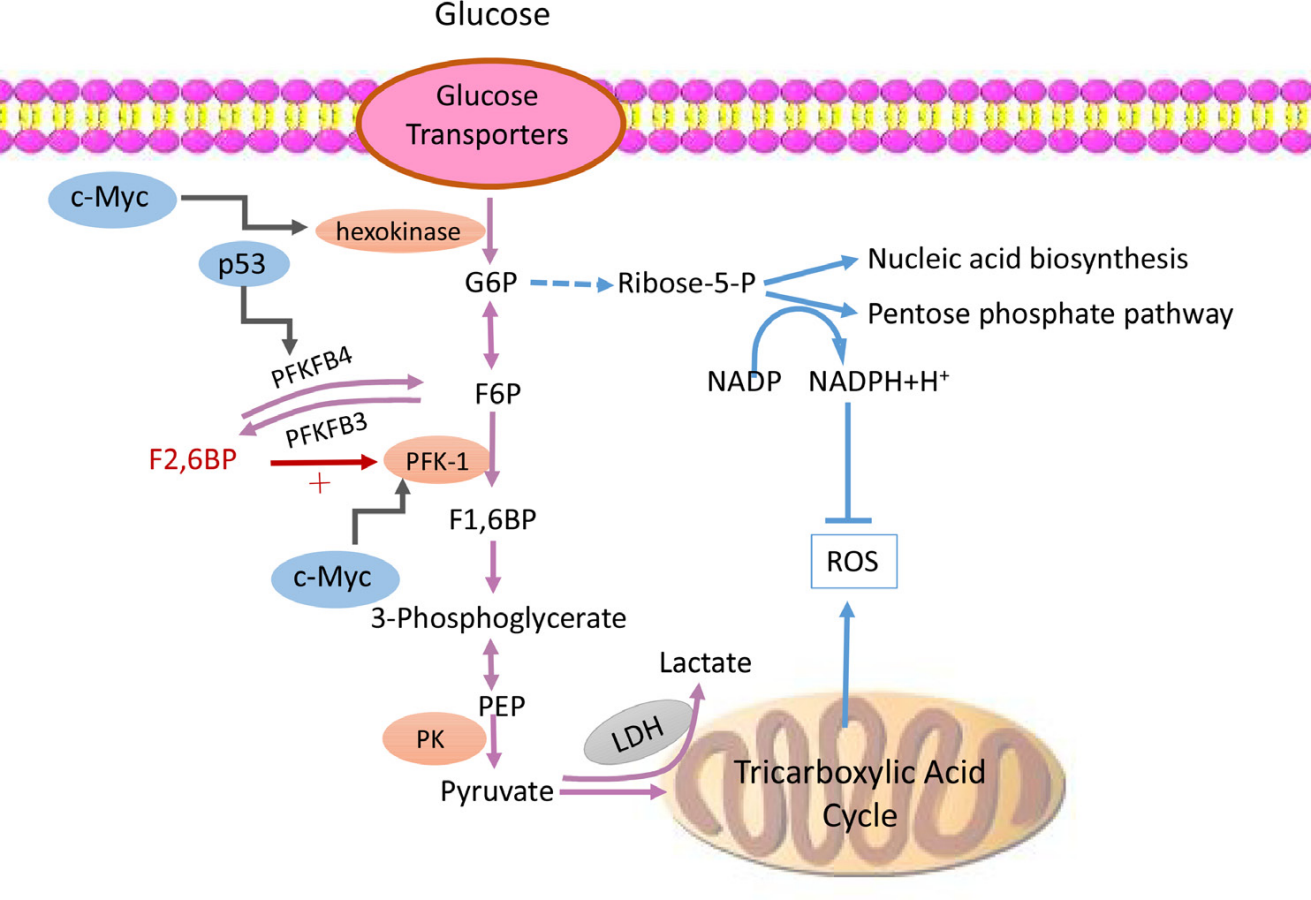
PFKFB3 participates abnormal glucose metabolic pattern in tumor cells Tumor cells maintain high-throughput glycolysis flux via variable mechanisms. Increasing glucose transporters and hexokinase activity result in increased intracellular concentrations of F2, 6BP and activated PFK-1. PFK-1 can be activated by F2, 6BP while it is not sensitive to be inhibited by increasing level of ATP, which leads to a sustained positive feedback. Meanwhile, PFKFB3 presents strong kinase activity and promotes the synthesis of F2, 6BP, which subsequently activates PFK-1 and upregulates glycolysis flux. Besides that, not only those lactic acid in mitochondrion will be transported out and be kept away from TAC, but also lactic acid located in cytoplasm is blocked by LDH and single carboxylate transport protein from re-entering the biological oxidation pathway. Finally, also a number of tumor-related genes, such as *MYC*, *HIF*, the *P53* and so on are involved in the formation of this abnormal metabolic pattern of tumor cells. Abbreviations: glucose-6-phosphate (G6P), fructose-6-phosphate (F6P),fructose-1, 6-bisphosphate (F1, 6BP), fructose-2, 6-bisphosphate (F2, 6BP),phosphofructokinase-1 (PFK-1), nicotinamide adenine dinucleotide (NAD), reactive oxygen species (ROS), lactate dehydrogenase (LDH), phosphoenolpyruvic acid (PEP), pyruvate kinase (PK), tricarboxylic acid cycle (TAC).

Increased glucose transporter (GLUT) and hexokinase activity are found in malignant cells, resulting in increased intracellular concentrations of F2, 6BP and activated PFK-1. PFK-1 must be activated by F2, 6BP because it is not sensitive to ATP inhibition, which leads to sustained positive feedback. This is a biochemical explanation for the high-throughput glycolysis maintained by tumor cells even in high ATP or normoxic environments. Furthermore, other mechanisms exist for malignant cells to promote or maintain anaerobic glycolysis (Figure 1). For example, lactic acid can also re-enter the biological oxidation pathway to produce more ATP, but lactate dehydrogenase and single carboxylate transport protein transport lactic acid out of the cell to block it from the TAC [24]. Another hypothesis is that the high concentration of lactic acid around the tumor cells is not simply the product of anaerobic glycolysis, but also important raw material for proliferation. This phenomenon is particularly likely to found in tissues and organs with excellent oxygen conditions, such as in airways [25]. Potentially, the tumor stromal cells could secrete lactic acid to satisfy the need of rapidly proliferating tumor cells. In this sense, tumors can be recognized as a parasitic disease [26, 27]. Therefore, an increasing number of researchers have focused on the relationship between tumorigenesis and the Warburg effect.

The abnormal metabolic pattern of tumor cell regulated by a number of important tumor-related genes, such as *MYC*, *HIF*, the *P53* gene, the PI3K (phosphatidylinositide 3-kinase)/AKT pathway, etc. has been widely accepted. *P53*^−*/*−^ cells have been found to have higher anaerobic glycolytic flux than wild-type cells, generating more lactate and presenting impaired mitochondrial respiratory functions [28]. This suggests that *P53* gene can inhibit glycolysis, and specifically downregulate GLUT, increase F6P, decrease F2, 6BP, inhibit lactate transporters, inhibit PDKs (pyruvate dehydrogenase kinases), induce mitochondrial oxidative regulators, synthesize cytochrome C oxidase 2, and compete with HIF-1 for limited transcriptional activators [29]. Recent published data has shown that *P53* genes with three mutated lysine mutations, *P53*^*3KR*^, lost the ability to block the cell cycle and regulate senescence and apoptosis, but still retained the ability to inhibit anaerobic glycolysis and reduce intracellular ROS. Surprisingly, *P53*^*3KR/3KR*^ mice were not observed to suffer with thymic lymphoma and die as early as *P53*^−*/*−^ mice [30]. The mice knockout experiment proved that *P53*′s ability to inhibit tumorigenesis is primarily due to its ability to control anaerobic glycolysis and decrease intracellular ROS. Carbohydrate levels, in turn, can also affect P53 levels. For instance, in *P53* mutated cells, downregulation of glucose levels can promote mutant *P53* deacetylation and degradation [31, 32]. *P53* can inhibit tumor growth by controlling anaerobic glycolysis, while *PFKFB3* can promote glycolysis. Therefore, *PFKFB3* inhibitor can be proposed as a therapeutic agent for *P53* mutant tumor cells [33].

### PFKFB3 expression in tumor cells

Based on the characteristics of the glucose metabolism of tumor cells and the kinase activity of PFKFB3, Kessler et al. analyzed the expression of PFKFB3 in 40 human astrocytoma and 20 normal brain tissue samples and found that PFKFB3 protein levels in high-grade astrocytoma were significantly increased compared to low-grade astrocytoma and the corresponding normal brain tissue, while PFKFB3 mRNA were not increased [34]. Further studies have shown that PFKFB3 mRNA was elevated in gastric cancer tissue compared to normal gastric tissue, as well as in gastric cancer cell lines (MKN45, NUGC3) [35], and in head and neck squamous cell carcinoma (HNSCC) compared with adjacent mucosal tissue [36]. In aggressive neoplasms, including colon, breast, ovarian, and thyroid original carcinomas, PFKFB3 expression was markedly increased [5]. PFKFB3 also plays an important role in the survival of acute myeloid leukemia (AML) cells, as PFKFB3 is a novel downstream substrate of the mTOR (mechanistic target of rapamycin) signaling pathway. Activating mTOR by HIF results in PFKFB3 expression upregulation, whereas knocking down PFKFB3 subsequently inhibits mTOR-activated AML cells and promotes their apoptosis [6]. These results contribute to the study of PFKFB3 inhibitors in tumors.

### PFKFB3 inhibitors application in tumors

#### In hematologic malignancies

The JAK2V617F (Janus kinase 2 V617F) mutation presents in the majority of patients with myeloproliferative neoplasms [37], researchers from the Dana-Farber Cancer Institute revealed upregulated PFKFB3 expression under hypoxic conditions and down regulated in response to Jak inhibitor application. Furthermore, STAT5 (signal transducer and activator of transcription 5), a known target of JAK2, was found inducing PFKFB3 expression. Although it is not known whether JAK2 was able to activate PFKFB3 expression directly, or through downstream effectors of STAT5, it can be speculated that PFKFB3 activation is required for JAK2 and STAT5. The outcome of this research suggested that targeting PFKFB3 may represent a novel therapeutic strategy in diseases associated with activated JAK mutations [38]. In both normoxic and hypoxic environments, PFKFB3 inhibition by compound or PFKFB3 gene knockdown decreases intracellular ROS dramatically and prevents the proliferation of tumor cells *in vitro* and *in vivo*. A similar situation was found in BCR-ABL-positive cells. As previously reported, KU812 and BaF3 cell lines presented higher levels of PFKFB3 and intracellular ROS when transfected with BCR-ABL, glucose uptake was found obviously increased simultaneously in both cell lines. When imatinib administrated, a significant decrease of glucose uptake was found in both transfected cell lines [39]. Besides the close association of the oncogene of hematologic disease and PFKFB3 expression, ROS enrichment, as a product of mitochondrial electron transport chain, was considered to results in risk of genetic instability, and is subsequently at risk of developing resistant tumor cells [40]. Therefore, it is speculated that PFKFB3 inhibitors are able to downregulate the glycolysis flux and decrease intracellular ROS, which helps to maintain genomic stability and prevent drug resistance and disease progression.

Brian Clem et al. [41] synthesized a small molecule PFKFB3 inhibitor 3-PO (3- (3-Pyridinyl) -1- (4-pyridinyl) -2-propen-1-one) in 2008, which blocked glucose uptake in Jurkat T cell leukemia cells and resulted in G2-M phase arrest, accompanied by a decrease in the intracellular concentration of F2,6BP, lactate, ATP, NAD+ and NADH. They also examined the effects of 3-PO on normal human bronchial epithelial (NHBE) cells and immortalized NHBE cells that transformed with telomerase (hT), large T antigen (LT), and H-ras^V12^ (hT/LT/ras cells). HT/LT/ras-transformed cells were found most sensitive to 3-PO among these cells. As a lower intracellular concentration of F2, 6BP was found in hT/LT/ras-transformed cells than NHBE cells, it was speculated that down regulated intracellular F2, 6BP sensitized cells to 3-PO. Similarly, the PFKFB3^+/−^ LT/ras-transformed fibroblasts, which expressed lower level of PFKFB3 protein and with lower concentration of intracellular F2, 6BP, were more sensitive to 3-PO than their wild-type genetic matched counterparts (PFKFB3^+/+^ LT/ras). Another convincing proof is that doxycycline-induced ectopic PFKFB3 expression in Jurkat T cells, increased intracellular F2, 6BP and conferred 3-PO resistance. HL-60 leukemia cell xenograft models of BALB/c athymic mice established by this group proved the efficacy of 3-PO. Tumors were significantly inhibited after the administration of this compound at a dose of 0.07 mg/g of 3-PO daily twice every 9 days, and all the mice were well tolerated. In 2013, this group further identified 73 3-PO derivatives and screened out a more powerful molecule inhibitor, PFK15 (1-(4-pyridinyl)-3-(2-quinolinyl)-2-propen-1-one), which displayed an approximately 100-fold greater activity against PFKFB3 than 3PO. PFK15 has a quinoline ring, which composes the ADP/ATP binding site of PFKFB3. PFK15 showed a lower IC_50_ (half maximal inhibitory concentration) than 3-PO (0.72 μm vs 5.57 μm) in Jurkat cells [42]. The combination of PFK15 and rapamycin was found to inhibit cell proliferation in AML cells [6].

#### In solid tumors

The efficacy of PFKFB3 inhibitors in solid tumors has also been demonstrated. PFK15 displayed better pharmacokinetic properties than 3-PO in Lewis lung carcinoma (LLC)-bearing C57Bl/6 mice, which had a marked reduction in clearance (46.2 mL/ min/kg vs 2312 mL/min/kg), increased T_1/2_ (5.1 h vs 0.3 h) and C_max_ (3053 ng/ml vs 113 ng/ml). Significant growth inhibition was observed in the LLC tumors after intraperitoneal administration at a dose of 25 mg/kg PFK15 every 3 days. Meanwhile, intracellular F2, 6BP was also found to be decreased, and caspase 3 cleaving within the tumor cells was revealed by immunohistochemistry. This outcome confirmed PFK15 was able to inhibit tumor growth and induce cell apoptosis *in vivo*. FDG-PET (fluorodeoxyglucose- positron emission tomography) imaging also proved that ^18^F-FDG uptake was reduced by 50% at 45 minutes after PFK15 administration on these tumor-bearing mice. PFK15 showed similar efficacy on the suppression of colon and pancreatic adenocarcinomas to irinotecan and gemcitabine, respectively. However, the activity of PFK15 against glioblastoma growth was lower than that observed by temozolomide [42]. The role of PFK15 was also validated in HNSCC, the pharmacological inhibition of PFKFB3 result in suppression of tumor growth and alleviation of the lung metastasis in the xenograft mice models [36]. In addition to tumor cell proliferation suppression and cell apoptosis inducing, PFK15 was also found inhibiting the invasion of gastric cancer cells through downregulating focal adhesion kinase expression and upregulating E-cadherin expression [43]. To evaluate the efficacy and safety of the PFK15-based synthetic compound, PFK158, phase I clinical trials (NCT02044861) were implemented in 2014, and no serious adverse events were reported during observation for almost one year.

Based on the efficacy of the glycolysis flux regulation of PFKFB3, PFKFB3 inhibitors were expected to work in drug-resistant tumor cells, or at least to combine synergistically with conventional drugs. Telang et al. [44, 45] revealed that in melanoma, breast cancer, and non-small cell carcinoma, PFKFB3 expression can be regulated by BRAFV600E, estradiol, and epidermal growth factor, respectively. Simultaneously inhibiting these oncoproteins or common ligands and PFKFB3 could cause a synergistic promotion in apoptosis and cytotoxicity of cancer cells *in vitro*. For instance, when BRAFV600E mutant melanoma A375 mice were divided into three groups, dramatic decrease of tumors to 50% or less was found in the combination treatment of vemurafenib and PFK158 group versus single agents BRAF inhibitor vemurafenib or PFK158 groups. In consideration of increased glucose uptake and lactate secretion trastuzumab resistant breast cancer cells exhibited, targeting PFKFB3 may also be an effective treatment against resistant HER2 (human epidermal growth factor receptor-2) positive breast cancers and may resensitize these tumors to anti-HER2 therapies. Julie et al. found that HER2 expression increased PFKFB3 expression and glucose metabolism and 3PO could significantly suppress HER2 positive breast cancer growth *in vivo* [46]. Taken together, these data demonstrated that PFKFB3 inhibitors may be able to universally overcome resistance to targeted cancer therapies. Furthermore, we predict that PFKFB3 inhibitors in combination with targeted cancer agents may improve response rates as well as progression-free survival.

PFKFB3 inhibitors have been proven to work as regulators for glycolysis and metabolism, which can be expected to suppress tumor cells *in vivo* and *in vitro* (Table 1). Of course, it is critical to explore whether PFKFB3 inhibitors affect the metabolism of normal cells and cancer stem cells (CSCs). Recently, Swedish researchers [47] found that PFKFB3 mRNA levels of breast CSCs and breast cancer cells *in vitro* were significantly higher than induced pluripotent stem (iPS) cells and human primary fibroblasts. Not only that, the transcription of PFKFB3 in CSCs were even higher than in tumor cells. A hypoxic environment induces PFKFB3 upregulation in CSCs, tumor cells, and iPS cells, but not in normal fibroblasts. Selective targeting of PFKFB3 by siRNA or 3-PO resulted in a significant decrease of extracellular lactate in breast cancer cells and a moderate decrease in iPS cells, but not in fibroblasts. Therefore, targeting PFKFB3 will exert only a tiny effect on the anaerobic glycolysis of normal cells. Additionally, PFKFB3 inhibitors triggered cell cycle arrest of breast cancer cells in G2 phase, suggesting that inhibition of PFKFB3 could prevent cancer cells from proliferation. While this phenomenon was not found in iPS and fibroblasts, which indicated that the efficacy of PFKFB3 inhibitor exert possibly only in malignant cells. Published data showed that transformed fibroblasts (PFKFB3^+/−^) were more sensitive to 3-PO than wild-type fibroblasts (PFKFB3^+/+^) [41]. These researchers revealed that inhibition of PFKFB3 could suppress glycolysis and induce G2 phase cell cycle arrest in cancer cells instead of iPS and fibroblasts. As no further evidence was found in other non-tumor cells, more data is required to confirm this speculation.

**Table 1:** Identified PFKFB3 inhibitors that induce cell proliferation suppression in tumor cells

Tumor types	Characteristics of tumor cells	PFKFB3 inhibitors	Synergetic agents	References
Acute myeloid leukemia	mTOR hyper-activated	PFK15	rapamycin	[6]
Acute lymphoblastic leukemia		3PO, PFK15		[40]
Head and neck squamous cell carcinoma		PFK15		[34]
Colon adenocarcinoma		PFK15		[40]
Pancreatic adenocarcinoma		PFK15		[40]
Glioblastoma		PFK15		[40]
Lung carcinoma		PFK15		[40]
Gastric cancer		PFK15		[41]
Breast cancer	ER^+^	PFK158	estradiol	[42]
Breast cancer	HER2^+^, resistance to trastuzumab	3PO	HER2 antagonist (lapatinib)	[44]
Melanoma	BRAFV600E mutant	PFK158	Vemurafenib	[43]

Abbreviations: mTOR : mechanistic target of rapamycin, HER2: human epidermal growth factor receptor-2, ER: estrogen receptor.

### Other biological functions of PFKFB3

#### Cell cycle regulation

**Figure 2:**
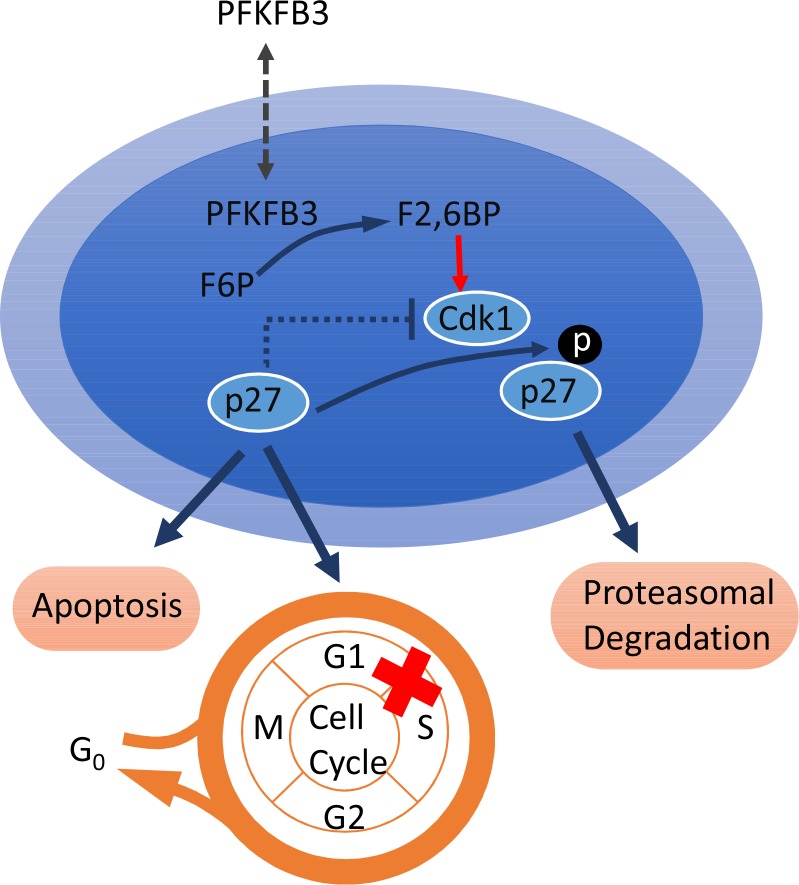
PFKFB3 participates in cell cycle regulation In the nucleus, F2, 6BP was induced by PFKFB3 and subsequently stimulated the phosphorylation of protein p27 at threonine 187 (T187) by Cdk, finally resulting in the ubiquitination and degradation of p27, which leads to cell cycle arrest at G1/S and increases apoptosis. Abbreviations: fructose-2, 6-bisphosphate (F2, 6BP), cyclin-dependent kinase (Cdk).

The role of PFKFB3 in tumorigenesis is mainly dependent on its function of regulating glycolysis, but recent studies have found that PFKFB3 is not only critical to the regulation of glucose metabolism in the cytoplasm, but also in regulating the cell cycle in the nucleus. Yalcin et al. revealed that PFKFB3 could be transported to the nucleus through a highly conserved nuclear localization motif in the C-terminal domain, stimulating cellular proliferation without affecting glycolysis. In the nucleus, F2, 6BP was induced by PFKFB3 and subsequently stimulated the phosphorylation of protein p27, which could potently block the G1/S transition and stimulate apoptosis at threonine 187 (T187) by cyclin-dependent kinase (Cdk), finally resulting in the ubiquitination and degradation of p27 [48] (Figure 2). Based on these mechanisms, siRNA knockdown of endogenous PFKFB3 was found to inhibit Cdk1 activity, which in turn stabilized p27 protein, leading to cell cycle arrest at G1/S and increasing apoptosis in HeLa cells, and this could be reversed by co-siRNA silencing of p27. Similarly, p27 protein expression was found to be increased when LLC cells were treated with 3-PO [49]. This indicates that PFKFB3 can enter the nucleus through Cdk1-mediated phosphorylation of p27 to promote cell cycle G1/S transition and suppress apoptosis, not only through regulation of glucose metabolism in the cytoplasm.

#### PFKFB3 and autophagy

**Figure 3:**
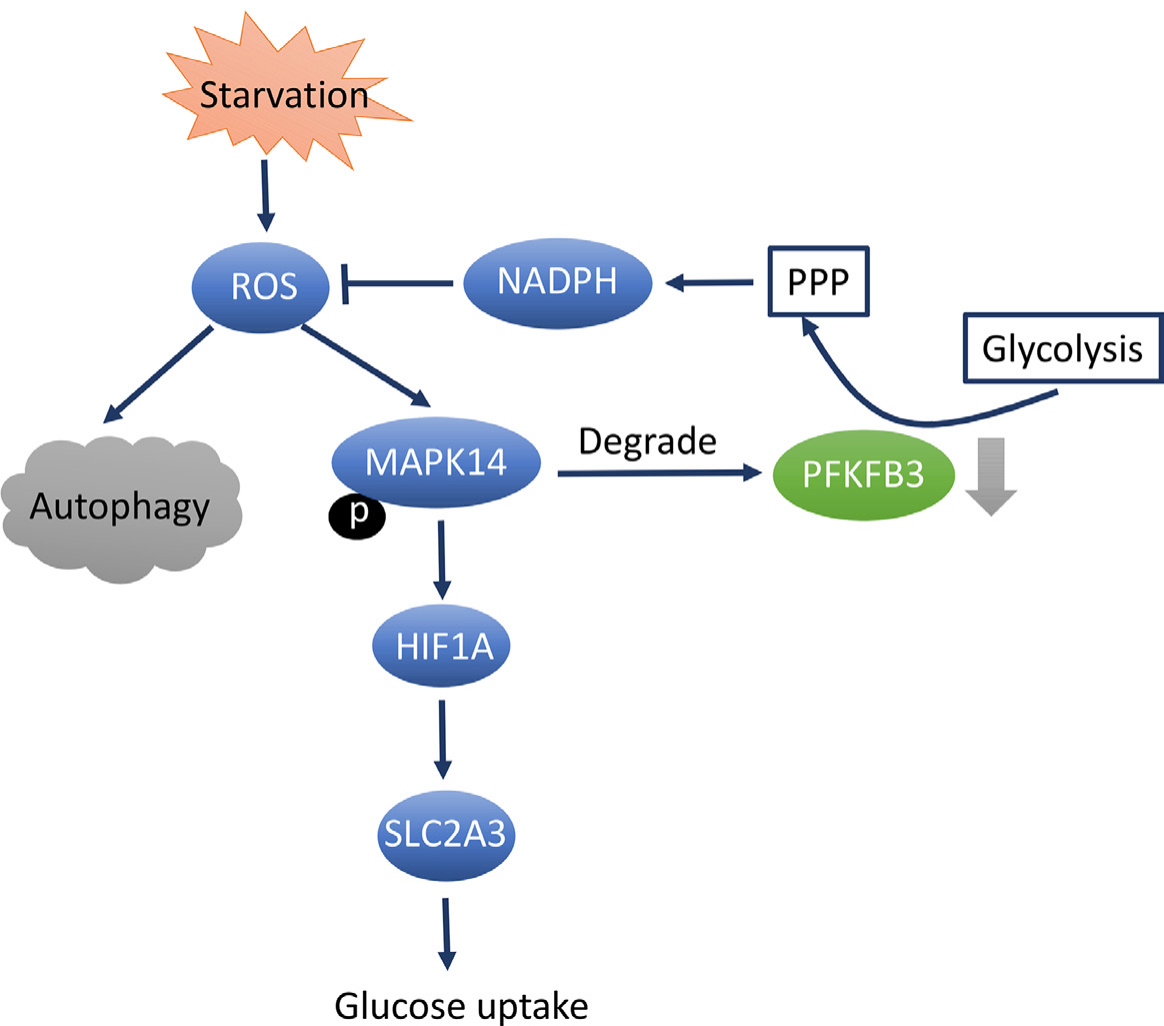
PFKFB3 inhibition results in autophagy downregulation through MAPK14-dependent manner Starvation causes activation of autophagy and MAPK14 phosphorylation. Active MAPK14 upregulates SLC2A3 expression through HIF1A stabilization and increases glucose uptake. In addition, MAPK14 induces a proteasome-dependent degradation of PFKFB3, which results in an increase of PPP at the expense of glycolysis. PPP enhancements fuels NADPH production, leading to reduced ROS levels and autophagy. Abbreviations: mitogen-activated protein kinase 14(MAPK14), Solute Carrier Family 2 (Facilitated Glucose Transporter), Member 3(SLC2A3), hypoxia-inducible factor (HIF), pentose phosphate pathway (PPP), reduced form of Nicotinamide adenine dinucleotide phosphate (NADPH), reactive oxygen species (ROS).

Autophagy is a pathway for the degradation of macromolecules and organelles, especially in stressful situations, such as hypoxia, malnutrition, protein misfolding, and pathogen infection. The proteins and organelles to be degraded are wrapped with double-membrane, then transported to the lysosome and phagocytosed. Cell structure, metabolism, and organelle renewal can be maintained by this process. This is also critical for cells to produce intracellular energy and transfer raw material to meet the demands of a changing environment. The initial research associating PFKFB3 and autophagy was in rheumatoid arthritis (RA), in which researchers found that T cells from RA patients failed to metabolize equal amounts of glucose as control cells, subsequently generated less intracellular ATP, and were prone to induce apoptosis. The researchers analyzed the expression of 29 glycolysis-related genes in activated CD4 T cells from RA patients and controls, found that PFKFB3 was the only gene transcript significantly different between the two groups. PFKFB3 expression was found to be significantly lower in T cells from RA patients, forced overexpression of PFKFB3 in RA T cells repaired the glycolytic insufficiency and the autophagic activity, subsequently protected cells from apoptosis. As NADPH levels were 50% higher in the RA-derived cells, indicating that G6P was shunted toward the pentose phosphate pathway (PPP), ultimately depleted the ROS of cytoplasm. In addition, when autophagy-specific marker LC3-II levels were measured in stimulated RA and control T cells, RA T cells were found failed to tap into cell-internal energy sources via autophagy. LC3-II levels were revealed a 30 to 40% reduction in the patient-derived cells. Correlated with PFKFB3 mRNA level, autophagy-related genes *LC3B* and *BECLIN-1* were also found to be significantly lower in RA T cells. The simultaneous appearance of glycolytic flux suppression and autophagic activity down regulation in RA T cells raised the question that whether they were mechanistically linked. Further western blot quantification and microscopic analysis of LC3-II confirmed this correlation, PFKFB3-specific RNA interference suppressed autophagy and forced PFKFB3 overexpression promptly accelerated autophagic activity. Therefore, it was proposed that PFKFB3-deficient T cells from RA patients entered PPP to generate more NADPH and less ROS, which in turn inhibited autophagy [50]. Enrico et al. analyzed HeLa and SK-BR3 cells in nutrient deprivation environments, they found that starvation conditions stimulated intracellular ROS and activated autophagy and mitogen-activated protein kinase 14 (MAPK14) phosphorylation. Activation of MAPK14 by HIF1A led to upregulation of glucose transporter SLC2A3 (solute carrier family 2 (facilitated glucose transporter), member 3) expression which subsequently increases glucose uptake. Additionally, MAPK14 induces proteasome-dependent degradation of PFKFB3, which shifts glucose metabolism from glycolysis to the PPP and inhibited autophagy. Instead, both the pharmacological inhibition and knockdown of MAPK14 will result in autophagy activation [51] (Figure 3). Though these data did not clarify how PFKFB3 regulated autophagy, the author initially put forward the conception that PFKFB3 play a critical role in regulating autophagy.

However, the relationship between PFKFB3 and autophagy remains controversial. Alden et al. found that PFKFB3 inhibition with either siRNA transfection or 3-PO in HCT-116 colon adenocarcinoma cells led to glucose uptake down regulation, ATP production reduction, ROS levels decreasing, and autophagy induction. As a resistance mechanism utilized by cancer cells to avoid being destroyed, autophagy stimulated by PFKFB3 inhibition may serve as a protective measure. It can be proposed that PFKFB3 antagonist, with an autophagy inhibitor, will be hopefully improve cytotoxic effects. Therefore, they assessed the cell death after application of a combination treatment of 3-PO and autophagy inhibitor chloroquine (CQ) and found that cell apoptosis was significantly increased. Additionally, researchers subcutaneously injected LLC cells into C57/BL6 mice, then treated them with different agents. The tumor mass was only significantly reduced in the group treated with a combination of 3-PO and CQ compared to the groups treat with a single agent [52]. Therefore, we propose the efficacy of PFKFB3 inhibitors as anti-cancer agents may be improved in combination with autophagy inhibitors. However, more unequivocal evidences for a regulatory role of PFKFB3 in setting the threshold for autophagy induction in different types of cells are required.

## SUMMARY

As a critical glycolysis regulator, the PFKFB3 gene/enzyme can influence cell cycle and tumor cell proliferation. PFKFB3 has high kinase activity, promotes F2, 6BP synthesis, and then activates PFK-1, increasing glycolysis for cell proliferation. Moreover, PFKFB3 can enter the nucleus to promote cell cycle transformation through Cdk1-mediated phosphorylation of p27 to regulate the cell cycle. The expression levels of PFKFB3 have been found to be higher in malignancies than in normal tissues and the suppressive function of PFKFB3 inhibitors on tumor cells has also been confirmed *in vivo* and *vitro*, suggesting that PFKFB3 may be a new target for tumor therapy and is expected to work in drug-resistant tumor cells. Even though the role of PFKFB3 in regulating the process of autophagy is unexpected and controversial, the combination of PFKFB3 inhibitors with autophagy inhibitors has been proven to be effective on certain tumor cells. In conclusion, recent studies have provided a new insight into the mechanism of PFKFB3-regulated cell proliferation and develop more promising anti-cancer therapy strategy. Further pre-clinical data of PFKFB3 inhibitors are required to clarify its potential application value for clinical use.
